# Hybrid Bioceramic: A Synergistic Platform for Structural Reinforcement and Bioelectric Stimulation Toward Smart Medical Devices

**DOI:** 10.1002/smll.202513459

**Published:** 2026-03-18

**Authors:** Sophia Selvarajan, Eunjung Byun, Naimur Rahman Niloy, Raneen Qasim, Junjira Tanum, Heejin Hwang, Seung Hyun Song, Geelsu Hwang, Albert Kim

**Affiliations:** 1 Department of Medical Engineering, College of Engineering, University of South Florida, Tampa, Florida, USA; 2 Department of Electrical Engineering, College of Engineering, Sookmyung Women’s University, Seoul, Republic of Korea; 3 Department of Preventive and Restoration Sciences, School of Dental Medicine, University of Pennsylvania, Philadelphia, Pennsylvania, USA

**Keywords:** barium titanate, implantable, piezoelectric, structural integrity, zirconia

## Abstract

As the rise of smart implants has led to a transformation in healthcare, the hybrid biomaterials have garnered significant attention due to the increasing demand for mechanical stability and functional properties. This study presents a barium titanate (BTO)-yttria stabilized zirconia (YSZ) bioceramic with varying compositions (between 40 wt.% and 60 wt.%), and sintering temperatures (1250°C–1450°C) to achieve clinically feasible structural integrity, piezoelectricity, mechanical strength, and a cellfriendly environment. By combining a piezoelectric phase with a mechanically robust ceramic phase, the hybrid system enables energy harvesting from physiological movements while maintaining long-term mechanical durability. The results demonstrate that both BTO and YSZ exhibit stable coexistence within a single material, segregated in a manner that preserves their respective structural and functional properties. Increasing BTO content enhances the piezoelectric coefficient (*d*_33_), while YSZ contributes to higher hardness and fracture toughness. Optimal sintering at 1350°C yields a dense microstructure that strikes a balance between electrical and mechanical performance. Furthermore, microstructural analysis via Raman spectroscopy, scanning electron microscopy, and X-ray diffraction confirms phase stability and controlled grain growth. The findings suggest that the BTO–YSZ bioceramic has strong potential for co-design of biofunctional and mechanically robust hybrid biomaterials, paving the way toward next-generation smart biomedical implants.

## 1 | Introduction

Since the successful clinical implementation of the pacemaker in the 1960s [1, 2], subsequent decades have witnessed a plethora of implantable medical devices (IMDs) due to numerous advancements in nano/microelectromechanical systems (N/MEMS), integrated circuits, wireless systems, and batteries [3–7]. Today, with the rapidly increasing number of drug-refractory diseases [8], an IMD is expected to transform the healthcare system more efficiently, proactively, and user-friendly.

Notable examples are orthopedic and dental implants, which are critical components in modern medicine. They are designed to restore function and aesthetics in patients with musculoskeletal and dental deficiencies [9–11]. The success of these implants heavily relies on the selection of appropriate biomaterials that can withstand physiological loads, resist corrosion, and integrate seamlessly with biological tissues. Particularly, they aim to closely mimic natural tissues and bones, including high mechanical strength, fatigue resistance, and biocompatibility for improved stability and longevity. Traditionally, metals like titanium and its alloys have been the materials of choice due to their excellent mechanical properties and favorable biological responses [12, 13]. Similarly, dental restoration materials require both aesthetic properties and mechanical robustness, presenting a unique challenge in biomaterial research. The current gold standard is zirconia-based ceramics, i.e., yttria-stabilized zirconia (YSZ), offering high strength, chemical stability, biocompatibility, and fracture toughness, with the added benefit of superior aesthetics (i.e., a tooth-like color). Studies have shown that zirconia implants exhibit comparable success rates to titanium [14]. Additionally, the application of nanotechnology in biomaterials has led to the creation of nanostructured surfaces that promote better cell adhesion and proliferation, thereby enhancing osseointegration [15].

Beyond orthopedic and dental applications, the principles of biomaterials also extend to the rapidly evolving field of smart implants, including neural interfaces and brain implants. The same challenges, i.e., power autonomy, structural durability, and biocompatibility, are even more critical when designing implants that interface directly with the nervous system. As neural prosthetics advance, integrating smart bio-electronic materials that can both harvest energy from physiological processes and interact dynamically with neural tissues becomes essential. In particular, piezoelectric and triboelectric materials, such as barium titanate (BaTiO_3_ or BTO), have shown potential for neural stimulation and regeneration [16, 17]. Note that BTO is renowned for its ferroelectric and piezoelectric characteristics, which are instrumental in converting mechanical stimuli into electrical potential. This transduction capability is pivotal for promoting osseointegration, as it emulates the natural electrical potentials generated during bone loading. Studies have demonstrated that BTO can enhance osteoblast differentiation and proliferation, thereby accelerating bone healing processes [17].

However, achieving an optimal integration of mechanical durability, bioelectrical activity, self-powered, and biocompatibility for a single biomaterial remains a key challenge in implant design. While BTO offers strong electromechanical functionality but limited load-bearing capability, YSZ provides exceptional fracture toughness and wear resistance, motivating the exploration of hybrid ceramic strategies. To address these complementary limitations, synergistic BTO–YSZ hybrid bioceramics have been explored as multifunctional systems capable of simultaneously supporting structural integrity and bioelectrical functionality. This bioceramic is anticipated to not only enhance structural integrity but also facilitate in vivo energy harvesting, that may potentially power implantable medical devices (IMDs) or stimulate cellular activities for improved healing. Recent in vitro studies have further demonstrated that when zirconia surfaces are functionalized with 3% – 5% BTO, bacterial growth may be inhibited [18, 19], suggesting dual functionality in promoting tissue integration and preventing infections.

Here, we present the next-generation BTO-YSZ hybrid bioceramic that aims to simultaneously retain and maximize multiple innate properties, including piezoelectricity, mechanical resilience, biological compatibility, and functional adaptability. To achieve this, we adopt an unconventional approach that preserves phase segregation, harnessing the tetragonal crystal structures of both components to maintain their intrinsic functionalities. The integration of the two phases enables simultaneous enhancement of structural stability and electromechanical functionality, while preserving the intrinsic advantages of each constituent through controlled phase segregation. Given the significance of composition ratio and sintering temperature, this study systematically investigates their influence on microstructure evolution, phase interactions, piezoelectric performance, and mechanical integrity. At an optimal sintering temperature of 1350°C, controlled interfacial reactions promote favorable grain growth and boundary interactions, sustaining a stable piezoelectric coefficient while enhancing mechanical durability through microstructural reinforcement. By fine-tuning these parameters, we establish a BTO-YSZ hybrid biomaterial as a promising nextgeneration multifunctional biomaterial, paving the way for smart orthopedic, dental, and neural implants that combine bioelectric stimulation with superior mechanical performance.

## 2 | Results and Discussion

### 2.1 | Microstructural Evolution With Sintering Temperature

The crystal structure of two materials plays a crucial role in determining their compatibility, mixing, and the formation of hybrid bioceramics. When YSZ is introduced into BTO at the nanoparticle level and sintered (both of which have a tetragonal crystal structure), the sintering profile governs crystal structure, leading to grain growth, phase stability, and overall microstructure. The detailed fabrication process and schematics are provided in the experimental section and in the Supporting Information (Figure S1). The sintering temperature alters the kinetics of grain boundary movement, densification behavior, and potential phase interactions, which dictate key structural and functional modifications. To retain the intrinsic functionalities of both BTO and YSZ, our approach is to maintain the coexistence of chemically distinct ceramic phases within the composite through controlled phase segregation, while promoting grain growth.

The body of literature indicates that the sintering process is well underway for both BTO and YSZ at lower temperatures (<1200°C), where they begin to form distinct grains and grow [20, 21]. For example, the onset of sintering (initial particle bonding) in BTO typically begins around 900°C–1000°C, and by ~1200°C, the material develops a clear polycrystalline grain structure, which then coarsens as the temperature increases. The upper temperature limit of BTO is around 1460°C, where it may undergo a structural transformation to a non-perovskite hexagonal phase [22]. For YSZ, full densification typically occurs around 1400°C–1500°C, but grain growth begins at ~1200°C. Sintering pure YSZ at 1200°C yields a partially dense structure (around 50%–60% of theoretical density), indicating that neck formation and grain boundaries have developed [23]. Based on the known sintering behavior of the individual components, the co-existence of these two phases via controlled phase segregation becomes feasible. Specifically, under a tailored sintering temperature, BTO can achieve near-full densification, while YSZ remains partially densified, preserving distinct microstructural domains. To systematically investigate and optimize this balance, we explored the impact of sintering temperature across a controlled range of 1250°C, 1300°C, 1350°C, 1400°C, and 1450°C. This temperature window was carefully selected to span the critical regime where BTO achieves optimal densification without undergoing high-temperature decomposition or phase instability, and where YSZ transitions from initial grain formation toward partial consolidation.

**FIGURE 1 |:**
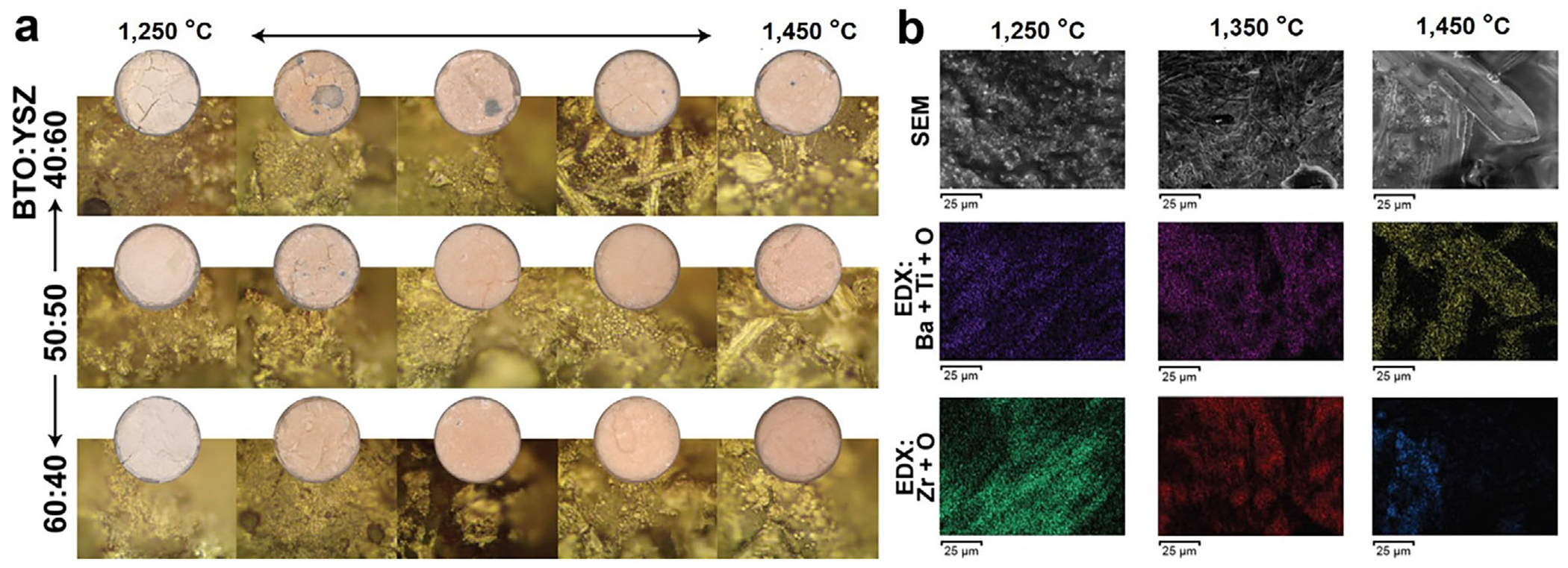
Evolution of BTO-YSZ hybrid bioceramic: (a) Evolution of the surface morphology observed by optical microscope, (b) segregation of BTO in YSZ grains observed by SEM and EDS.

Figure 1 presents the evolution of surface morphology, grain growth, and elemental distribution in BTO-YSZ hybrid bioceramics across sintering temperatures, as characterized by optical photography, microscopy, scanning electron microscopy (SEM), and energy-dispersive X-ray spectroscopy (EDS) analysis. At 1250°C, insufficient densification resulted in a porous, rough surface with prominent intergranular voids (Figure 1a), while SEM images revealed restricted diffusion and fine-grained microstructures pinned at grain boundaries (Figure 1b). As the temperature increased to 1300°C – 1350°C, the bioceramic exhibited markedly improved microstructural uniformity. YSZ nanoparticles, which segregate preferentially at BTO grain boundaries, act as robust grain growth inhibitors by elevating the activation energy for boundary migration, thereby moderating grain coarsening without entirely arresting it. This controlled grain evolution yielded a smoother, denser surface architecture, as confirmed by optical microscopic images and EDX mapping. This result aligns with other studies that showed even small amounts (e.g., ~ 1%) of YSZ can effectively control grain size, enhance uniformity, and contribute to improved mechanical integrity [24].

Despite previous reports suggesting barium zirconate titanate (BZT) formation via BTO-YSZ sintering [25–27], our data reveal clear phase segregation: BTO maintained distinct rod-shaped grains, with dimensions smaller than those observed in pristine BTO (Figure S2), suggesting minimal compositional interdiffusion. Concurrently, densification behavior, summarized in Figure S3, corroborates the microstructural trends. At 1250°C, the bioceramic exhibited low densification (density = 4.06 ± 0.251 g/cm^3^) and high porosity, undermining mechanical stability. Sintering at 1350°C substantially improved densification (4.61 ± 0.326 g/cm^3^), enhancing mechanical integrity while preserving functional surface for potential interactions with biological or environmental interfaces. At 1450°C, however, near-complete densification (5.09 ± 0.092 g/cm^3^) was accompanied by excessive BTO grain growth, as the grain boundary inhibition effect of YSZ diminished due to enhanced atomic diffusion. The emergence of exaggerated grain facets and potential hexagonal phase formation at this temperature risked compromising piezoelectric functionality and fracture toughness.

**FIGURE 2 |:**
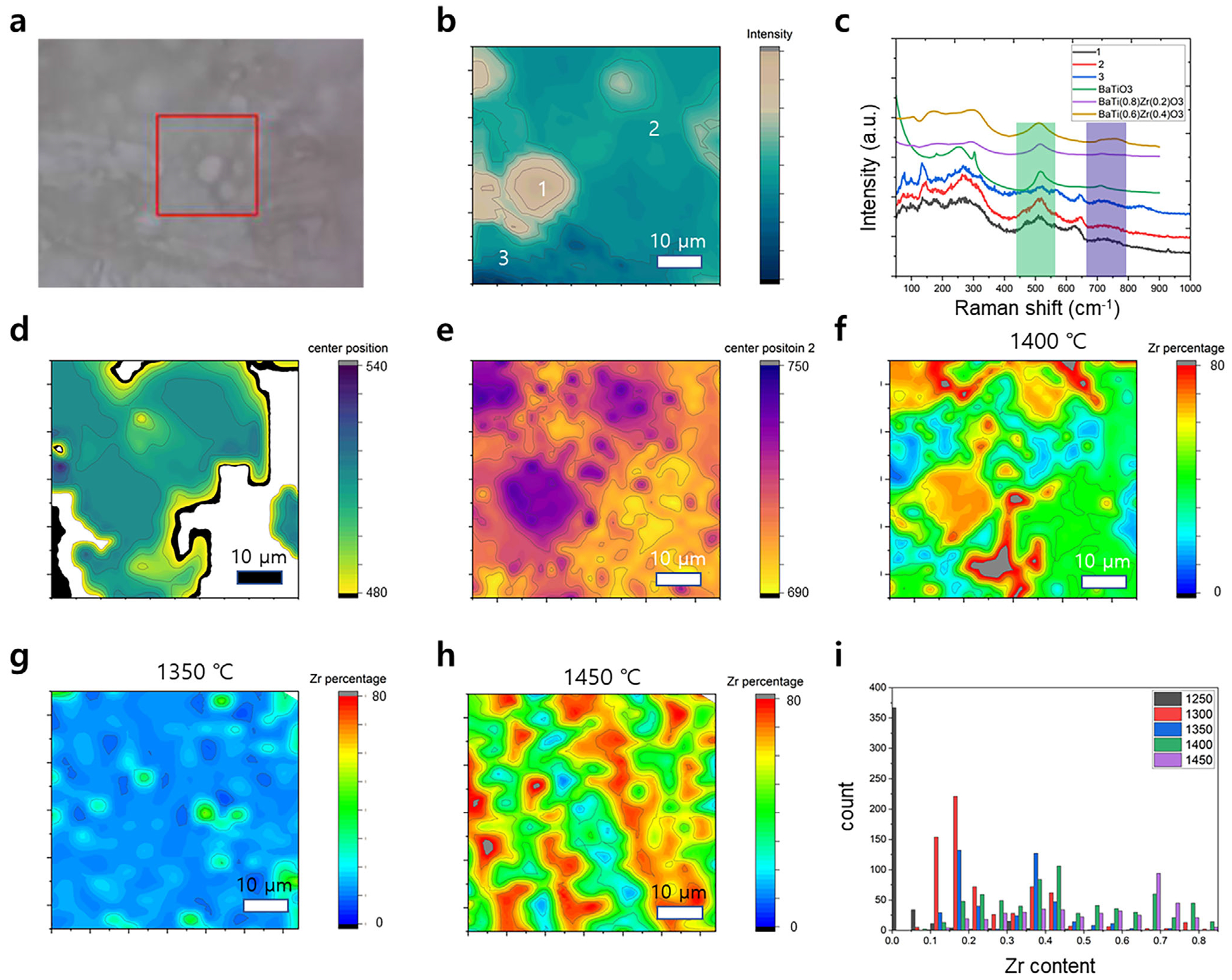
Characterization of Zr diffusion via Raman Spectroscopy: (a) Optical photo of the sample surface (red box indicates the mapping region), (b) Intensity mapping of A1 mode, (c) representative Raman spectra with the reference spectra of BTO and BaTi_1–x_(Zr_x_)O_3_, (d, e) Peak center position mapping result, (f–h) predicted Zr contents mappings for the samples sintered in 1400° C, 1350°C, and 1450°C, respectively, (i) Histogram of Zr percentage for the samples sintered in 1250°C–1450°C.

While the evolution of BTO-YSZ bioceramic with the increasing temperatures suggests that a high sintering temperature is essential for obtaining high mechanical and flexural rigidity, the high sintering temperature also promotes atomic diffusion of Zr into BTO, which is known to destabilize the piezoelectric tetragonal phase [25–27]. Across the bioceramic sintered at various temperatures, we observed boundary regions that showed both Ba and Zr elements in the EDS analysis, which indicates possible secondary phases. As such, we employed Raman spectroscopy to further characterize the chemical composition and phases of the BTO-YSZ bioceramic, aiming to further determine the optimal sintering temperature (Figure 2).

Figure 2a shows the optical photograph of the 60 wt.%:40 wt.% sample sintered at 1400°C, where the textured surface is visible (note that there is a positional offset between the scan area illustrated by the red box and the actual scan area). The intensity mapping of the A_1_ LO mode (from 710 to 770 cm^−1^) is shown in Figure 2b, where distinct regions with clear boundaries are observed. Three representative Raman spectra taken at three different positions (as denoted in Figure 2b) are shown in Figure 2c along with the reference spectra of BTO and BaTi_1-x_(Zr_x_)O_3_ [27]. Compared to the pure BTO reference spectrum, the point spectra taken from the sample also show broadening and peak position shifting of A_1_(LO) and A_1_(TO).

These peak broadening and shifting are related to Zr incorporation within the BTO, as established by the prior literature [25–27]. The two prominent BTO or BZT-related modes (A_1_(TO) near 510 cm^−1^ and A_1_(LO) near 710 cm^−1^ shift under the incorporation of Zr ions within BTO perovskite [28]. The TO mode phonon redshifts due to the larger ionic radii of the Zr^4+^ ion compared to the Ti^4+^ monotonically, while the behavior of the LO mode phonon is non-monotonic, likely due to the emergence of an additional peak within the region. For the quantitative analysis of Zr diffusion percentage, we employed the following methodology: first, the peak center positions for both A_1_(LO) and A_1_(TO) were obtained by independently fitting Lorentzian peaks using custom Python code. The center position maps of A1(TO) and A1(LO) are shown in Figure 2d,e, respectively. We can see that both peak positions show a degree of correlation. We note that the white regions in Figure 2d are where the single Lorentzian fitting was unsuccessful due to the presence of additional peaks arising from YSZ (we note that the fitting results with R^2^ values less than 0.9 were discarded).

Using the peak fitting results obtained from prior literature data, the Zr contents are estimated via a weighted k-nearest neighbor algorithm (please refer to Supporting Information for a detailed discussion on the analysis) from the center positions of both A1 mode phonons (we note that the accuracy of the estimation is around 10% and larger when Zr content is low). The resulting Zr percentage (as in x in BaTi_1–x_(Zr_x_)O_3_) mapping is shown in Figure 2f. As can be seen, the Zr migration within the BTO matrix is significant, ranging from 0.1 to above 0.8, which could be highly detrimental to the piezoelectric properties of the bioceramic. In addition, the segregation of Zr-rich and Ti-rich regions can be clearly distinguished within the BZT regions. On the other hand, Zr migration into BTO was relatively low up to the sintering temperature of 1350°C, as shown in Figure 2g. At a higher temperature, the Zr-rich regions further segregate into rectangular grains (Figure 2h), indicating the phase transition of BZT from the tetragonal phase. The results suggest that above 1350°C, interdiffusion between BTO and YSZ becomes more active, promoting solid-state reactions at the grain boundaries that lead to the formation of secondary phases such as BaZrO_3_, TiO_2_-rich regions, or BaO_5_TiZr (BZT).

The histogram data (Figure 2i) also clearly shows the extent of migration and the co-existence of BTO, YSZ, and BZT. The critical temperature at which BZT emerges was between 1350°C and 1400°C, with dependency on the mixing ratio between BTO and YSZ, consistent with the prior report of 1320°C ~ 1350°C being the critical temperature [29]. The presence of BZT phases (with Zr content > 0.2) is expected to degrade the piezoelectric response by disrupting the perovskite lattice structure of BTO, altering its dielectric constant, and reducing ferroelectric polarization.

**FIGURE 3 |:**
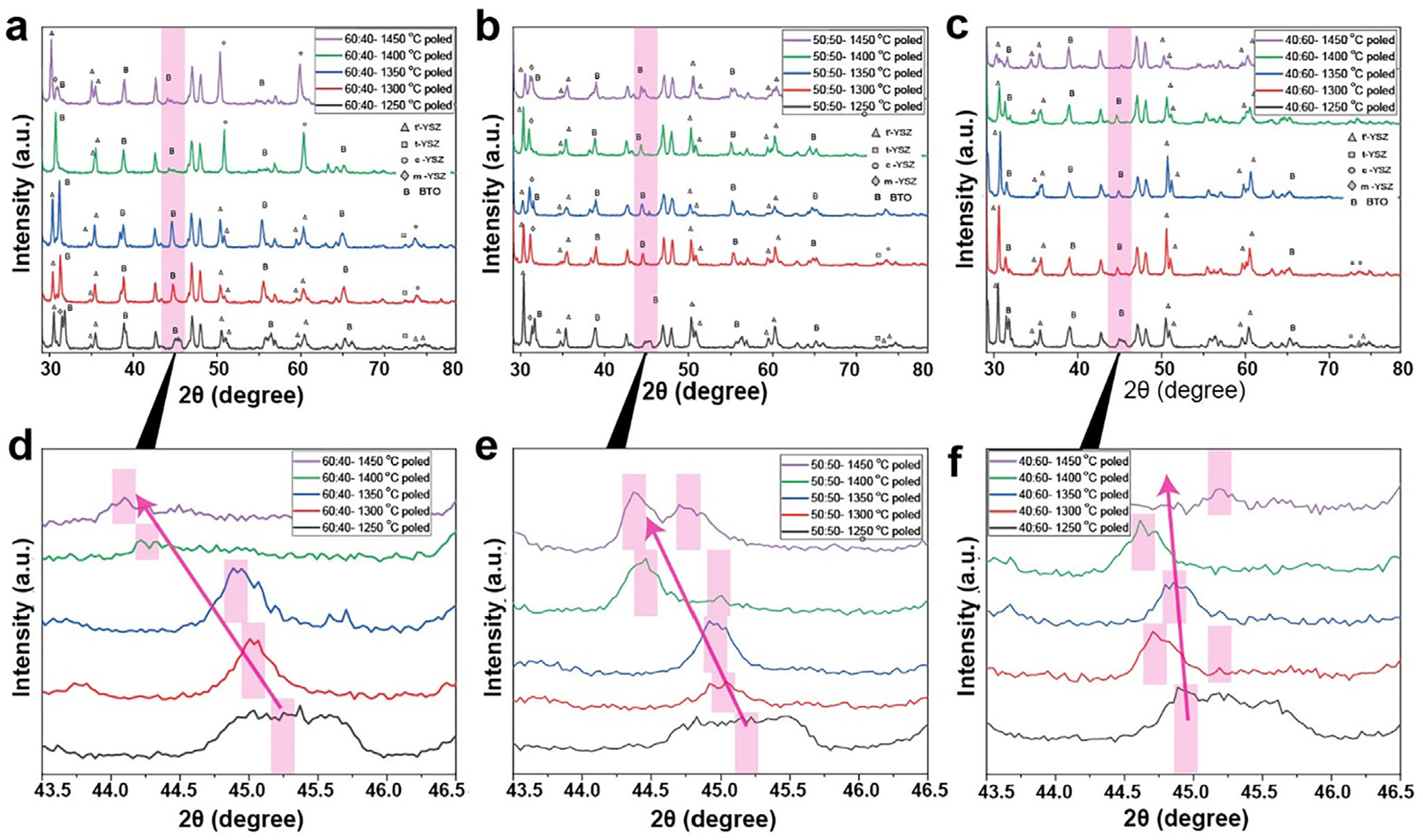
X-ray diffraction (XRD) patterns of BTO-YSZ bioceramics with varying composition ratios (60:40, 50:50, 40:60) and sintering temperatures. (a–c) Full-range XRD spectra of poled samples sintered at 1250°C, 1300° C, 1350°C, 1400° C, and 1450° C for compositions (a) 60:40, (b) 50:50, and (c) 40:60, respectively. Peaks are indexed to tetragonal YSZ (t-YSZ, △), cubic YSZ (c-YSZ, □), monoclinic YSZ (m-YSZ, ◊), and BTO (B). (d–f) Enlarged view of the diffraction region near 2*θ* ≈ 44°–45.5°, highlighting the evolution and shifting of peaks associated with BTO phase and crystallographic texture across sintering temperatures for (d) 60:40, (e) 50:50, and (f) 40:60 compositions. The pink shaded regions and arrows indicate key peak shifts and intensity changes with temperature, suggesting crystallographic transitions and phase evolution.

The X-ray diffraction (XRD) results shown in Figure 3 further support the crystalline phases, particularly with regard to BZT formation. The peak splitting of the tetragonal (200)/(002) reflections in BTO progressively shifts with increasing sintering temperature, indicating an increase in lattice distortions due to interfacial interactions. At 1350°C, Raman data indicate the formation of a marginal secondary phase, which preserves the tetragonal structure of BTO and maintains its intrinsic piezoelectric response. Accordingly, the XRD data of the same 60 wt.%:40 wt.% samples shown in Figure 3a indicate that the peak near 45.2° (corresponding to BTO tetragonal splitting) exhibits a leftward shift (−0.2°) with increasing sintering temperature, indicating lattice expansion due to Zr^4+^/Y^3+^ substitution and interfacial strain, with no major secondary phase formation detected. When YSZ is mixed with BTO, Zr^4+^ and Y^3+^ may substitute for Ti^4+^ or occupy interstitial sites in the BTO lattice. Since ZR^4+^ (0.72°A) or Y^3+^ (0.90°A) are larger than Ti^4+^ (0.605°A), this substitution causes lattice expansion, increasing the d-spacing between atomic planes, leading to lattice expansion and increasing the d-spacing between atomic planes [30]. Note that the reaction kinetics are insufficient to drive significant phase transformations at 1250°C, preserving the structural stability of the BTO-YSZ bioceramic with limited interdiffusion and phase segregation.

### 2.2 | Composition and Sintering Temperature Influence on Piezoelectric Performance and Mechanical Strength

After validating that the sintering temperature determines the overall fusion of BTO and YSZ, we explored the impact of the composition of each element, specifically on the piezoelectric properties. Theoretically, if YSZ remains primarily as a segregation phase and does not react significantly with BTO, the piezoelectric coefficient (*d_33_*) remains relatively stable. However, we witnessed that excessive phase migration and secondary phase formation occurred, which could lead to a reduction in dielectric permittivity, consequently compromising the overall piezoelectric performance. This creates a trade-off: excessive YSZ can lead to excessive grain boundary segregation, disrupting the formation of ferroelectric domains, whereas an optimized 3YSZ addition can successfully control grain growth while maintaining a reasonable balance between mechanical strength and electrical performance.

Paired with the microstructural evolution, variations in BTO/YSZ ratios lead to distinct piezoelectric behaviors. Compositions with higher BTO content (e.g., 60 wt.%:40 wt.%) exhibit an enhanced ferroelectric domain density; as seen in Figure 3a, the XRD of BTO/YSZ at a 60:40 ratio, sintered at 1350°C, clearly shows tetragonal splitting around (200)/(002). It translates to a higher piezoelectric coefficient (*d*_33_). However, excessive phase migration promotes the formation of secondary phases, such as BaZrO_3_, TiO_2_-rich regions, or BaO_5_TiZr (BZT), at higher sintering temperatures (e.g., 1400°C–1450°C), which reduces domain wall mobility and limits electromechanical coupling. An intermediate composition (50 wt.%:50 wt.%), shown in Figure 3b, provides an optimal balance between piezoelectric performance and mechanical robustness. Conversely, higher YSZ concentrations (e.g., 40 wt.%:60 wt.%) suppress piezoelectric activity due to the dilution of ferroelectric phases, while also restricting excessive grain growth. Evidently, the XRD results show that the (200)/(002) peaks at 1450°C have disappeared (Figure 3c). The piezoelectric performance in relation to the sintering temperature at the same composition also varied. For example, 50 wt.%:50 wt.% samples sintered at 1350°C exhibit the highest dielectric constant (*d_33_* = 13 pC/N), attributed to optimized domain wall mobility. At lower sintering temperatures (1250°C – 1300°C), the BTO perovskite phase may have been preserved; however, its reduced grain size and limited domain wall motion result in the lower piezoelectric coefficient (*d_33_* = 9 pC/N on average). At higher temperatures (1400°C–1450°C), the secondary phases impede domain activity, reducing overall piezoelectric performance and exhibiting 10 pC/N.

The combined Raman and XRD analyses enable a direct structure–property interpretation of the observed piezoelectric trends. Raman mapping reveals that Zr incorporation within the BTO lattice spans a broad compositional range, from low substitution levels (x ≈ 0.1–0.2) to highly Zr-rich regions (x > 0.5 and locally approaching >0.8), while XRD confirms progressive lattice expansion and suppression of tetragonal peak splitting with increasing sintering temperature and YSZ content. At lower Zr incorporation levels (x ≲ 0.2), the tetragonal perovskite structure is largely preserved, enabling effective ferroelectric domain formation and alignment during poling, which supports higher *d*_33_ values. In contrast, higher Zr incorporation reduces lattice tetragonality, weakens long-range ferroelectric order, and introduces compositional disorder, as evidenced by Raman peak broadening and XRD peak merging, leading to suppressed domain-wall mobility. These Zr-rich regions are weakly ferroelectric or non-ferroelectric at room temperature and are inefficiently poled under the applied electric field, thereby reducing the effective ferroelectrically active volume. Consequently, *d*_33_ increases up to the optimal sintering window (≈1350°C), where densification and domain development dominate, but decreases at higher temperatures and/or higher YSZ content as Zr-rich phases become more prevalent and interconnected, quantitatively linking the structural evolution to the degradation of piezoelectric performance.

**FIGURE 4 |:**
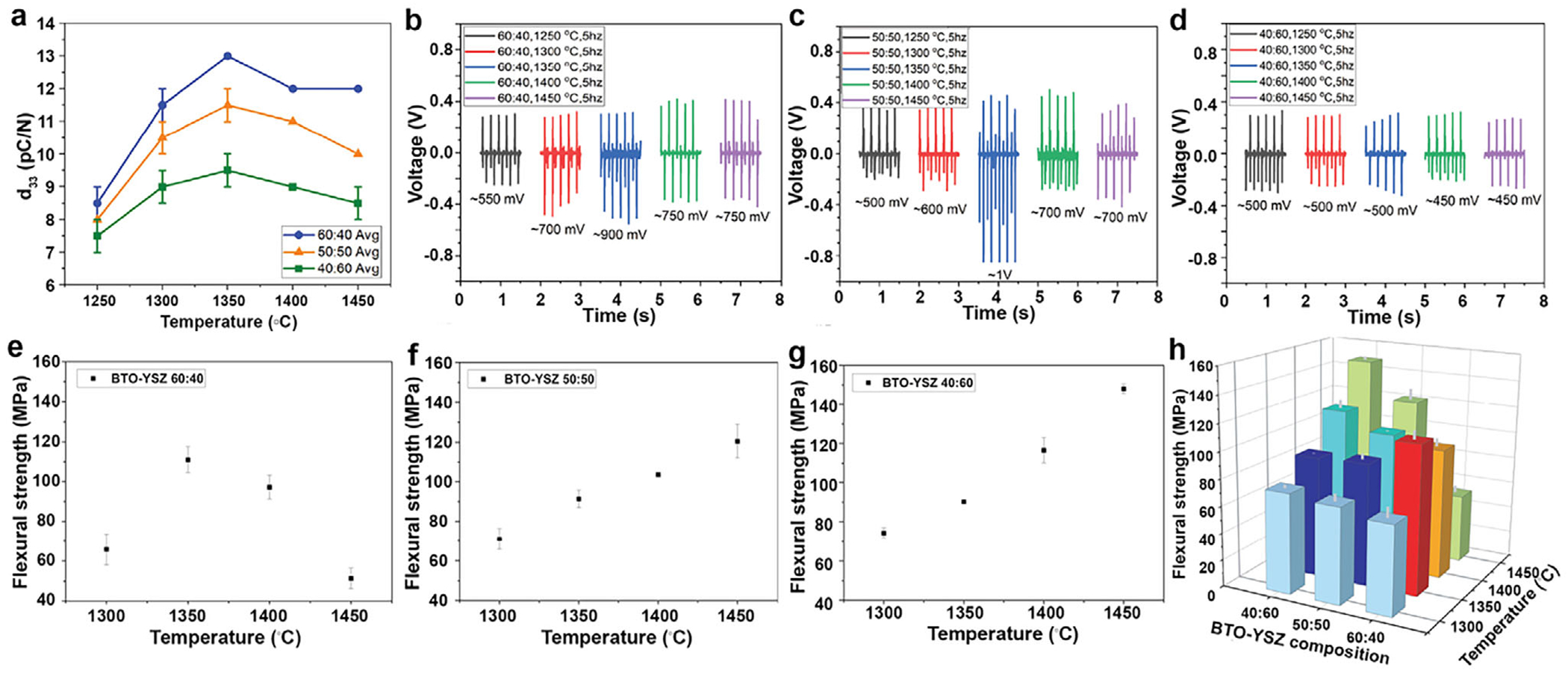
Piezoelectric performance and Flexural strength of the BTO–YSZ bioceramics: (a) Measured *d*_*33*_ ranges across sintering temperature and composition. Summary of electric potential generation under 5 Hz vibration excitation as a function of sintering temperature for (b) 60 wt.%:40 wt.%, (c) 50 wt.%:50 wt.%, and (d) 40 wt.%:60 wt.% compositions. The flexural strength (average ± SD) for (e) 60 wt.%:40 wt.%, (f) 50 wt.%:50 wt.%, and (g) 40 wt.%:60 wt.%. (h) 3D bar graph comparing the flexural strength of BTO–YSZ bioceramic across different sintering temperatures and compositions.

We corroborate the measured piezoelectric coefficient (*d_33_*) with various mechanical stimulations, including a vibration table (Eisco Labs; VBGN) and a thumb press. Overall, we observed various electrical potential generation of the bioceramic due to the combined effect of sintering temperature and compositional ratio. The measured *d_33_* values ranged from 7 to 13 pC/N (Figure 4a). The bioceramics were subjected to variable cyclic frequencies (i.e., 1, 5, and 10 Hz), and their corresponding opencircuit voltage (Voc) was recorded. An increasing trend in Voc under higher frequency is observed (Figure S4). The highest voltage output was achieved at 10 Hz, while the lowest was recorded at 1 Hz, indicating the frequency-dependent electrical response of the bioceramic. At a moderate frequency of 5 Hz, which is a frequency commonly found in human biomechanics [31], the 60 wt.%:40 wt.% bioceramic sintered at 1350°C exhibited a peak voltage of 900 mV, followed by a slight decline at higher sintering temperatures (Figure 4b). This peak voltage output corresponds to a sintering window where optimal grain growth, densification, and domain mobility result in a well-balanced microstructure. This behavior is consistent with prior reports on BTO-based piezoelectric ceramics, which exhibit maximum electromechanical response at intermediate sintering temperatures (∼1300°C–1350°C), followed by performance degradation at higher temperatures due to grain coarsening, phase instability, and over-densification [26]. Additionally, the electric potential generated from the thumb press (Figure S5) exhibits the ability of the bioceramic to harvest energy from low-frequency motions, such as chewing and biting, which can be utilized in smart dental implants for therapeutic applications.

Figure 4c shows the electromechanical response of 50 wt.%:50 wt.% bioceramic. Despite having a lower BTO content than the 60 wt.%:40 wt.% composition, it displayed the highest output overall, with 1.0 V from the bioceramic sintered at 1350°C, suggesting a balanced phase composition and optimized domain wall mobility, resulting in enhanced electromechanical response. This composition simultaneously maintains high voltage output while exhibiting improved mechanical integrity, as discussed in Section 2.3, without a pronounced reduction in piezoelectric performance. Conversely, the 40 wt.%:60 wt.% bioceramic exhibited a consistently lower voltage output across all sintering temperatures, with voltage outputs stagnating around 500 – 560 mV and dropping further to 450 mV from the higher sintering temperatures (> 1400°C), as presented in Figure 4d. The reduced voltage output correlates with the higher YSZ fraction and the associated reduction in effective piezoelectric domain activity, consistent with the lower *d*_33_ values observed for this composition compared to the 60 wt.%:40 wt.% and 50 wt.%:50 wt.% bioceramics.

Based on the electrical potential results, the piezoelectric performance of BTO-YSZ bioceramic is a function of both composition and sintering temperature. A higher BTO content enhances *d_33_* values but risks grain growth-induced degradation. The optimal bioceramic was found to be a 50 wt.%:50 wt.% composition sintered at 1350°C, which enables the best trade-off between densification, grain structure control, and domain wall activity, leading to superior piezoelectric properties.

The mechanical strength and surface morphology of BTO-YSZ bioceramics are critical for their suitability in structural applications, particularly in load-bearing and self-powered functional implants [18, 32]. The sintering parameters directly influence the hardness and fracture resistance of the bioceramics, while surface morphology governs environmental interactions and potential degradation behavior. From a mechanical standpoint, YSZ inclusion enhances fracture resistance through its high intrinsic hardness and phase-segregated distribution within the BTO matrix, improving the mechanical durability of the bioceramic. At the nanoparticle level, YSZ-rich regions at grain boundaries can impede crack propagation and modify local stress distributions, contributing to improved fracture resistance. Accordingly, crack deflection is considered a possible contributing toughening mechanism, inferred from the observed phase-segregated microstructure and established reports on YSZ-mediated mechanical reinforcement, rather than being directly demonstrated in the present study [23, 24, 33]. Achieving the optimal balance between mechanical integrity and piezoelectric functional performance is therefore governed by both the composition and sintering temperature.

The mechanical performance of the BTO–YSZ bioceramics was evaluated through a three-point bending test using a miniaturized specimen geometry. Although fabrication initially targeted 20 mm-long bars in accordance with ASTM C1161 Type B specifications, the reliable sintering of these larger specimens with high aspect ratios proved challenging due to intrinsic material limitations and process-induced stresses. Specifically, the heterogeneous microstructure of the BTO–YSZ bioceramic, combined with differences in thermal expansion coefficients and sintering kinetics between the two phases, resulted in differential densification along the bar. These factors, amplified over the longer specimen length, frequently resulted in microcracking, warping, and non-uniform grain growth, thereby compromising mechanical reliability. Moreover, the probability of critical flaw formation increases with specimen volume in brittle ceramics, such that larger specimens are statistically more likely to contain strength-limiting defects [34]. Conversely, miniaturized specimens typically exhibit a reduced flaw population, which can result in higher apparent flexural strength values compared to standard-sized bars [35]. Accordingly, the flexural strength values reported here should be interpreted as comparative indicators for evaluating the influence of composition and sintering temperature, rather than as absolute ASTM-compliant strength values. To address these limitations and ensure homogeneous densification with minimized residual stresses, smaller bars with dimensions of 3.5 × 3.0 × 7.0 mm^3^ were fabricated. This miniaturization not only mitigated internal defect generation but also enabled accurate and reproducible mechanical testing under a customized three-point bending setup tailored for small-scale specimens. The flexural strength (FS) was calculated using the standard formula:

(1) $$FS=\frac{3FL}{2bh^{2}}$$

where *F* is the maximum applied load at fracture (N), *L* is the span length (mm), *b* is the specimen width (mm), and *h* is the specimen height or thickness (mm).

Load and displacement data were recorded using a 100–1000 electromechanical universal testing machine (Test Resources Inc., Shakopee, MN, USA), equipped with a standard three-point bending fixture. The machine applied a load at a constant crosshead speed until fracture occurred. The obtained flexural strength values were used to assess the influence of zirconia content on the mechanical performance of the bioceramic.

As discussed in Section 2.1, the lower sintering temperatures (1250°C – 1300°C) exhibit incomplete densification and rough surface morphology in the material, which causes higher porosity and reduced mechanical strength. A smoother, well-densified surface enhances fracture resistance and mechanical reliability, whereas a rough, porous morphology introduces stress concentration points that may lead to crack initiation under mechanical loads. The 60 wt.%:40 wt.% bioceramic (Figure 4e) demonstrated the highest flexural strength of ∼116 MPa at 1350°C, followed by a significant decrease to 51 MPa at 1450°C. Grain coarsening and loss of phase stability occurred above 1400°C, which results in weakened grain boundary cohesion and diminished strength in BTO-rich ceramics [36]. Additionally, exaggerated grain growth occurs at higher sintering temperatures, resulting in internal stress concentrations leading to reduced fracture toughness [37]. At 1350°C, there is the optimal balance between densification and grain size, resulting in minimal porosity and strong grain interlocking.

The 50 wt.%:50 wt.% composite (Figure 4f) demonstrated a consistent increase in flexural strength, rising from 71 MPa at 1300°C to 121 MPa at 1450°C. This increase is attributed to the stabilizing effect of YSZ, which inhibits excessive grain growth and provides microstructural reinforcement that may promote crack deflection, thereby contributing to higher flexural strength. In contrast, the 40 wt.%:60 wt.% composites (Figure 4g), which contain the highest concentration of YSZ, exhibit the highest overall flexural strength at 167 MPa with the 1450°C sintering parameter. The strong mechanical performance results from improved structural densification, minimal porosity, and zirconia-induced toughening, which helps resist crack propagation [36]. The flexural strength trend in the 40 wt.%:60 wt.% composition contrasts with that of the 60 wt.%:40 wt.%, reinforcing the dominant mechanical role of YSZ at higher concentrations. The composites show a temperature-dependent increase in flexural strength, peaking between 1350°C and 1450°C. Although the 60 wt.%: 40 wt.% composition reaches peak strength at 1350°C before experiencing thermal degradation, the 50 wt.%:50 wt.% composite shows steady improvement across the temperature range, while the 40 wt.%: 60 wt.% composition gains the greatest benefit from high-temperature sintering as well as a higher YSZ concentration (Figure 4h). While YSZ is widely recognized for enhancing fracture toughness in ceramic systems, direct fracture toughness (K_1_c) measurements were not performed in this study. Accordingly, mechanical durability is discussed based on flexural strength trends, microstructural observations, and established literature on zirconia-based ceramics, where fracture resistance is often inferred in the absence of direct K_1_c testing [23, 24, 36, 40]. Quantitative K_1_c evaluation will be explored in future studies.

### 2.3 | BTO-YSZ Biomaterial-Mediated Self-Powered Light Source for Phototherapy

**FIGURE 5 |:**
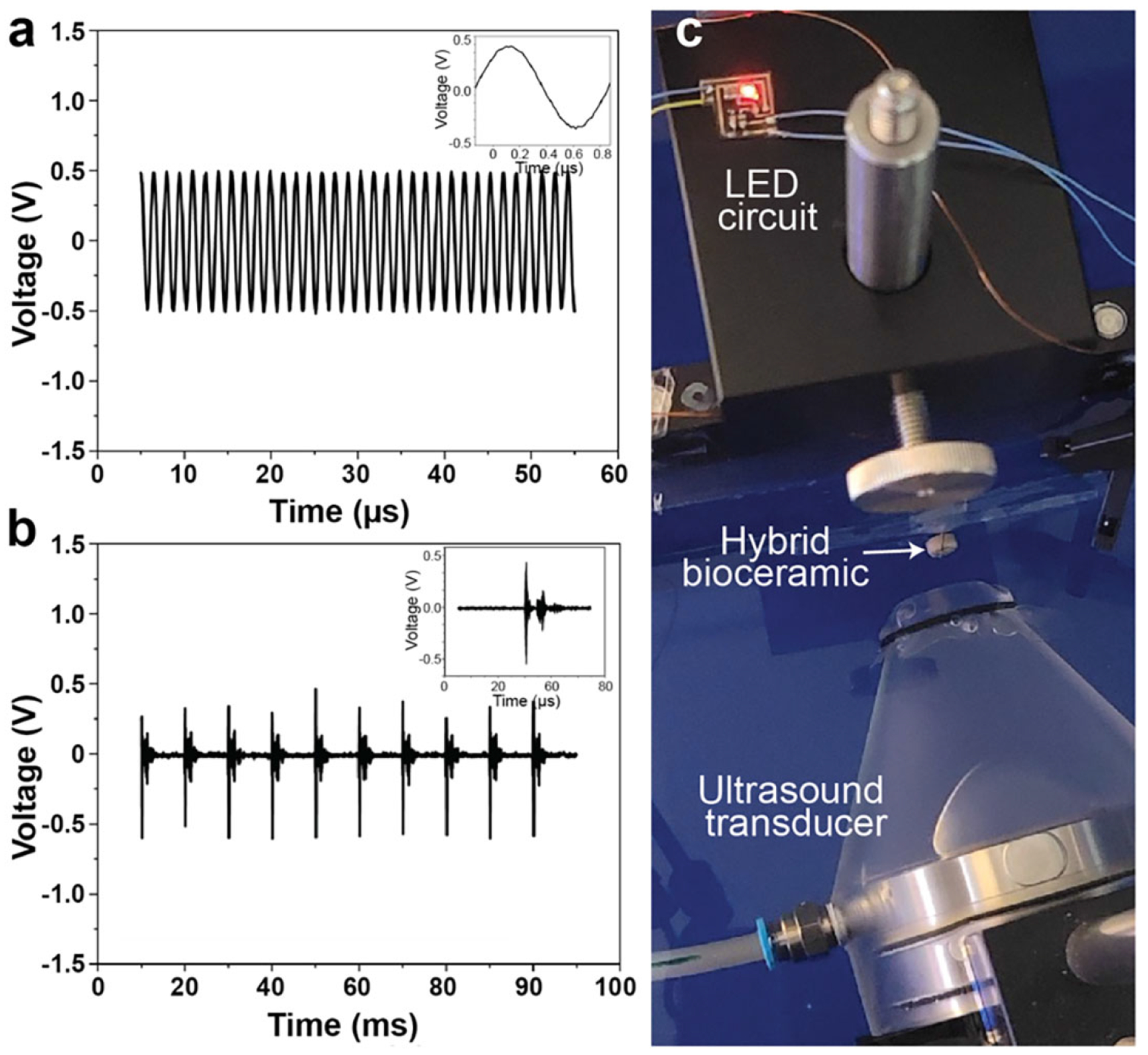
BTO –YSZ biomaterial-mediated self-powered light source for phototherapy. Output voltage generated from the BTO –YSZ composite (50 wt.%:50 wt.%) sintered at 1350°C, under (a) continuous-mode ultrasonic excitation and (b) pulsed-mode ultrasonic excitation. (c) Experimental setup for biomaterial-mediated self-powered light source showing the ultrasonic transducer aligned with the hybrid composite disc. The top inset highlights the red LED lit by the piezoelectric output under continuous-mode excitation. A full-wave bridge rectifier and capacitor circuit (not shown) was used to convert the AC output to DC for storing and powering the LED.

Based on our previous effort in enabling phototherapy in various applications [31] we implemented the BTO-YSZ bioceramic as a self-powered light source. It represents a promising avenue for next-generation load-bearing and self-powered functional biomedical implants, particularly in cancer treatment (Photodynamic Therapy), oral tissue regeneration [18, 38–40] or wound healing. To assess the energy-harvesting performance, a BTO – YSZ bioceramic disc (50 wt.%: 50 wt.%) sintered at 1350°C was exposed to ultrasound, with the transducer directly aligned to the hybrid disc surface. As shown in Figure 5a, continuousmode ultrasonic excitation (*f*_resonant_ = 670 kHz, Intensity = 17.73 mW/cm^2^) produced a stable sinusoidal voltage output with ∼ 1.1 V_p-p_. This response highlights efficient electromechanical coupling behavior of the composite, yielding consistent AC output suitable for rectification, energy storage, and self-powered applications, such as a light source. Figure 5b illustrates the composite’s response under pulsed ultrasonic excitation (*f*_resonant_ = 670 kHz, no. of cycles = 10, pulse period = 1 ms). While peak voltage levels were comparable, the output was intermittent and decayed rapidly between bursts, characteristic of pulsed operation. This can be advantageous in implantable systems where thermal management and power availability are critical.

To demonstrate the functional utility of the harvested energy, the rectified output under continuous excitation was used to drive a low-voltage red LED (*λ* = 630 nm; APHHS1005SURCK, King-bright), as shown in Figure 5c. A full-wave bridge rectifier (863-NSR1030QMUTWG, Onsemi) paired with a smoothing capacitor effectively converted the AC output into a DC voltage (bottom inset), enabling the LED to illuminate without an external power source. The top inset image confirms successful LED activation during ultrasound exposure, showing the composite’s capability as a self-powered light source. The LED output was quantified using a calibrated photodetector (model 53373, Edmund Optics; responsivity 0.34 A W^−1^ at 630 nm), yielding an estimated optical power of ∼70 μW and a corresponding irradiance of ∼7 mW cm^−2^. This irradiance lies within the low irradiance range reported for photobiomodulation and soft-tissue regeneration (typically < 50 mW/cm^2^), with therapeutic efficacy governed primarily by the delivered radiant exposure rather than irradiance alone [18, 41–43].

Beyond conventional load-bearing dental applications, the BTO–YSZ bioceramic shows strong potential as a self-powered therapeutic dental implant capable of harvesting masticatory forces to generate localized electrical output. This piezoelectric response may be utilized to drive implant integrated therapeutic functions, such as low-intensity LED-based phototherapy for gingival tissue healing and mitigation of peri-implantitis, without reliance on external power sources [18, 41–46].

### 2.4 | Biocompatibility and Cell-Biomaterial Interaction

The biocompatibility of novel biomaterials intended for oral applications is of critical importance. Ceramic biomaterials such as BTO and YSZ are widely recognized for their excellent biocompatibility, due to their chemical inertness, stability, and resistance to corrosion in physiological environments. When combined in a hybrid ceramic system, these materials form a multifunctional interface that supports cellular interactions while maintaining structural and chemical stability. However, the biocompatibility of their combination in a composite system remains less understood due to the added complexity of material interactions.

**FIGURE 6 |:**
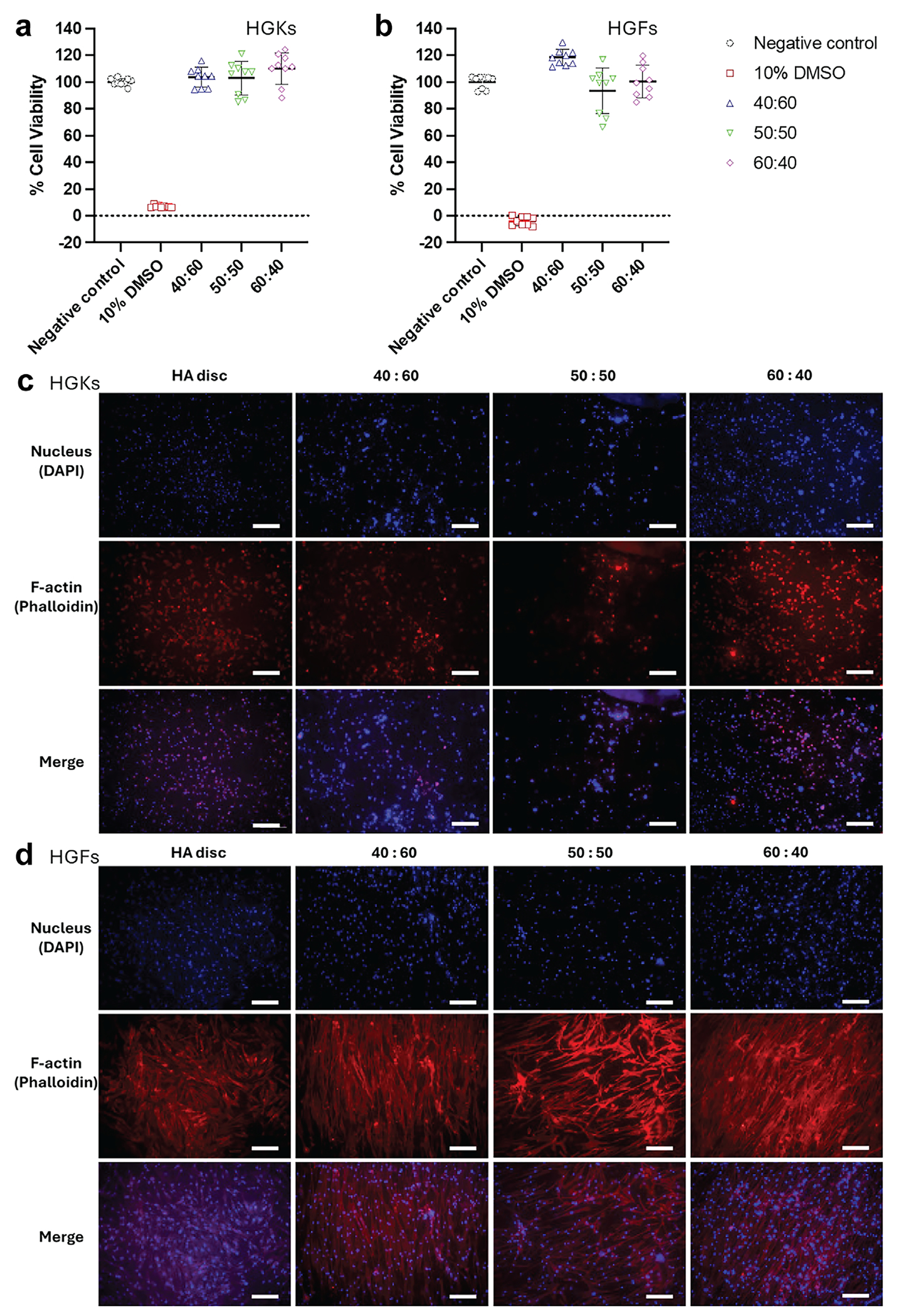
Biocompatibility and cell-biomaterial interaction. Cytotoxicity test of BTO-YSZ composites with a) human gingival keratinocytes (HGKs) and b) human gingival fibroblasts (HGFs). The tests were conducted three times on each sample, with three replications per test. Error bars are standard deviations (n = 3). Fluorescence images of c) HGKs and d) HGFs adhesion onto BTO-YSZ at different mixing ratios. Scale bar is 200 μm.

To investigate the cytocompatibility of BTO-YSZ composites with human oral tissues, extract-based cytotoxicity assays were performed using human gingival keratinocytes (HGKs) and human gingival fibroblasts (HGFs), following ISO 10993–5:2009 guidelines. MTT assays demonstrated that extracts from BTO-YSZ bioceramic at varying mixing ratios did not significantly affect the viability of either HGKs or HGFs compared to the untreated controls (Figure 6a,b). In contrast, the 10% DMSO cytotoxic control resulted in a dramatic reduction in cell viability. These findings indicate that both BTO and YSZ, individually and in combination, exhibit in vitro biocompatibility, supporting their potential for further biomedical development.

Beyond toxicity, cell adhesion is another critical parameter influencing the integration of biomaterials with host tissues. To assess this, we cultured HGKs and HGFs directly on BTO-YSZ bioceramic surfaces. The data revealed that both cell types successfully adhered to colonize the composites across all tested ratios, with performance comparable to hydroxyapatite (HA) control discs (Figure 6c,d). Notably, HGFs exhibited aligned growth patterns on BTO-YSZ bioceramic surfaces, likely influenced by parallel grooves introduced during surface polishing, which were absent on HA discs. This anisotropic cell orientation was not observed in HGKs. The comparatively lower apparent cell density observed on the 50:50 BTO–YSZ surface is attributed to enhanced cell spreading and alignment induced by composition-dependent surface microtopography, rather than reduced cytocompatibility, as supported by comparable cell viability across all compositions (Figure 6a,b). Previous studies have shown that surface topography, including microgrooves, pits, and ridges, can influence fibroblast morphology, orientation, and even differentiation [47–49]. Our results are consistent with these observations, suggesting that the microarchitectural features of BTO-YSZ bioceramic may enhance gingival fibroblast organization and attachment.

Altogether, the BTO-YSZ bioceramic demonstrated excellent cytocompatibility and supported favorable cell adhesion and orientation, particularly for fibroblasts, highlighting its promise for dental and craniofacial applications. The biocompatibility evaluation in this study was designed as an initial cytocompatibility screening using 24 h MTT viability and cell adhesion assays, consistent with first-stage biological evaluation recommended by ISO 10993–5 and commonly adopted for ceramic and piezoelectric biomaterials [50–53]. While these results confirm the absence of acute cytotoxicity, comprehensive long-term biological assessment, including extended in vitro and in vivo inflammatory response studies, will be conducted in future studies to advance clinical translation.

## 3 | Conclusion

Collectively, this work demonstrates that precise tuning of the sintering window and composition is essential, as the sintering temperature has a profound impact on simultaneously optimizing the microstructural evolution, functional performance, and mechanical robustness of BTO-YSZ bioceramic. From a broader perspective, this work establishes a robust framework for developing multifunctional bioceramics that integrate mechanical, electrical, and biological properties. The systematic approach to composition optimization and processing parameter control provides a valuable methodology that can be extended to other piezoelectric biomaterial systems. The successful demonstration of energy harvesting capabilities combined with biocompatibility represents a significant step toward the development of next-generation smart implants that can actively participate in therapeutic processes while maintaining structural integrity in load-bearing applications. Future developments should focus on in vivo validation, long-term stability assessment, quantitative fracture toughness (K_1_c) evaluation, and integration strategies for practical implant systems, building upon the solid foundation established by this comprehensive materials characterization study.

## 4 | Experimental Section/Methods

### 4.1 | BTO-YSZ Bioceramic Materials and Sample Preparation

BTO-YSZ composites were fabricated using 500 nm BTO nanoparticles (US research nanomaterials) and 100 nm 3 mol% yttria-stabilized zirconia (Sigma–Aldrich). An appropriate ratio of BTO nanoparticles and 3 mol% yttria-stabilized zirconia was dried at 100°C in a vacuum oven (AccuTemp-09, Across Intl.) for 1 h to remove any moisture. The mixture was mixed thoroughly using a rolling mixer (LC-3DMixer, 3D SYSTEMS) and transferred to a wax resin mold to obtain the corresponding disc shape. We then compressed the mixture using a compression machine to increase overall integrity and density. The wax mold with BTO nanoparticles was placed inside a muffle furnace (GSL-1700X, MTI Corp.) for sintering at desired temperature levels (i.e., 1250°C – 1450°C) for 180 min with a ramp of 5°C/min. At such a high temperature, the wax mold completely evaporates, leaving behind a well-sintered, disc-shaped hybrid composite material. A similar protocol was followed for making 100% BTO and 100% YSZ discs. After sintering, the discs were polished to reduce surface roughness. For electrical connections, the conductive silver epoxy coating was used as the electrode material applied on both sides. The piezoelectric dipole moments were aligned for maximum piezoelectric output by poling the devices at 110°C by applying a high voltage of ∼ 2.2 kV/mm to a thickness of 1 mm in a silicone oil bath. External leads for voltage measurements were established using copper wires.

### 4.2 | Scanning Electron Microscopy (SEM) / Energy Dispersive X-Ray Spectroscopy Analysis

Scanning electron microscopy (SEM) was conducted using a JEOL JSM-IT210 system to observe surface morphology and microstructural features of the sintered BTO–YSZ bioceramic discs. Prior to imaging, the samples were sputter-coated with a 5 nm platinum layer to enhance conductivity and minimize charging effects. Elemental composition and distribution were analyzed using an energy-dispersive X-ray spectroscopy (EDS) system integrated with the SEM. EDS mapping was performed to assess the spatial distribution of Ba, Ti, Zr, and Y elements within the bioceramic matrix, particularly across grain boundaries and interfaces.

### 4.3 | Density Measurements

The bulk density of BTO–YSZ discs was measured using Archimedes’ method. Each measurement was repeated three times for accuracy, and the average density value with standard deviation was reported. The bulk density of BTO–YSZ composite discs was measured using the Archimedes method in distilled water at room temperature. Dried sintered discs were first weighed in air and then submerged using a suspension wire to obtain their apparent mass in water. The density (*ρ*) was calculated using the relation:

$$\rho =\frac{W_{\text{air}}}{W_{\text{air}}-W_{\text{water}}}\times \rho_{\text{water}}$$

*W*_air_ and *W*_water_ represent the sample’s weight in air and water, respectively, and *ρ*_water_ is the density of water at room temperature (0.998 g/cm^3^). Measurements were conducted on three replicate samples per group, and the mean density was plotted against sintering temperature (1250°C–1450°C) for each composite ratio (60:40, 50:50, and 40:60 BTO–YSZ).

### 4.4 | X-Ray Diffraction (XRD) Analysis

X-ray diffraction (XRD) analysis was performed using a Bruker D2 Phaser benchtop diffractometer (M-XRD) equipped with Cu K*α* radiation (*λ* = 1.5406 Å), operating at 30 kV and 10 mA. Scans were collected over a 2*θ* range of 20° to 80° with a step size of 0.02° and a scan rate of 2° /min. Phase identification was carried out by comparing the BTO–YSZ composites with reference patterns from the International Centre for Diffraction Data (ICDD) database. Peak splitting in the tetragonal BTO phase and shifts due to Zr/Y substitution were analyzed to infer lattice distortion and interfacial interactions.

### 4.5 | Raman Spectroscopy Analysis

Raman spectra were collected using XperRam S Raman spectrometer (Nanobase) equipped with a 532 nm excitation laser. The laser power was kept below 1 mW to prevent local heating. Mapping was performed over selected regions of the bioceramic surface. Spectral deconvolution and peak fitting were conducted using Lorentzian profiles to extract A1(TO) and A1(LO) modes using custom Python code. Zr substitution and phase evolution were quantitatively estimated using a weighted k-nearest neighbor algorithm based on the Raman shift trends of BaTi_1–x_(Zr_x_)O_3._

### 4.6 | *d_33_* Analysis

The piezoelectric charge coefficient (*d*_33_) of sintered and poled BTO–YSZ composite samples was measured using a PK-D3-F10N force transducer integrated with a commercial *d*_33_ meter system (PolyK/Bruker; model 90–2030.1), which includes a shaker head, *d*_33_ display unit, and force measurement module. The setup applies a sinusoidal mechanical force of approximately 0.25 N at 110 Hz and directly reads the induced charge per unit force in picocoulombs per Newton (pC/N).

Prior to measurement, each sample was poled at 110°C in a silicone oil bath by applying a DC field of ∼2.2 kV/mm for 15 min, followed by cooling under constant field. Samples were then cleaned, dried, and mounted between the shaker head probes. A light clamping force was applied using the threaded adjustment knob, and the measurement was performed after the display stabilized.

The system was calibrated using a certified standard sample with a known *d*_33_ value of 409 pC/N. Polarity checks were performed by flipping the sample 180° to ensure consistent and symmetric measurements.

The Poling conditions were optimized using dense 100% BTO by varying electric field strength and duration (Figure S6). As shown in Figure S6a–c, increasing the field from ~1 to ~2.2 kV mm^−1^ and extending the poling duration from 15 to 30 min progressively enhanced the piezoelectric output from ~2.5 to ~9 V_p–p_, indicating improved ferroelectric domain alignment. Based on this trend, ~2.2 kV mm^−1^ for 20 min was selected as the optimized poling condition, as higher electric fields resulted in electrical shorting and unstable polarization behavior. Such poling fields and durations are widely used for BaTiO_3_-based and related piezoceramics and represent standard practice for achieving stable domain alignment without inducing electrical failure [54–56]. Comparison of unpoled and poled samples under low-frequency excitation (Figure S7) shows negligible output in the unpoled state (Figure S7a) and a pronounced, stable voltage response after poling (Figure S7b), confirming effective activation of piezoelectric functionality. The optimized poling parameters were subsequently applied uniformly to all BTO–YSZ composite samples to enable a fair and direct comparison of composition- and sintering-dependent effects. While composition-specific optimization of polarization conditions may further enhance absolute piezoelectric performance, maintaining constant poling parameters in this study isolates the influence of microstructural evolution and phase interactions. Exploration of composition-dependent poling strategies will be pursued in future studies.

### 4.7 | Mechanical Strength Measurements

Mechanical properties were evaluated by performing three-point bending tests on miniaturized bar-shaped specimens (3.5 mm × 3.0 mm × 7.0 mm). The flexural strength was calculated using the formula *σ* = 3FL/(2bh^2^), where F is the fracture load (N), L is the span length (mm), b is the width, and h is the height of the specimen. The tests were conducted on a universal testing machine (Model 100–1000, Test Resources Inc.) with a constant crosshead speed of 0.5 mm/min. The results were averaged from three replicates per group, and standard deviations were reported.

### 4.8 | Low-Frequency Energy Harvesting

To evaluate the piezoelectric energy harvesting capability of the BTO–YSZ hybrid composites under low-frequency mechanical stimulation, a custom experimental setup was developed using a commercial vibration generator (Eisco Labs, VBGN) mechanically interfaced with the composite sample. This configuration was designed to simulate low-frequency cyclic forces relevant to biomedical implants and wearable energy-harvesting applications.

The vibration generator was driven by a sinusoidal waveform generated directly from a Keysight DSOX3034G digital oscilloscope equipped with a built-in waveform generator. A 20 mV peak-to-peak sine wave was applied at excitation frequencies of 1, 5, and 10 Hz to emulate quasi-static and dynamic loading conditions. The signal was subsequently amplified using an external amplifier to ensure sufficient mechanical actuation of the hybrid composite. Input voltage and frequency were continuously monitored and verified using the oscilloscope interface.

The amplified signal was delivered to the actuator platform, which was integrated into a custom-designed test fixture. The fixture enabled direct mechanical contact between the actuator and the BTO–YSZ composite discs. A spring-loaded compression mechanism ensured uniform contact and axial force transmission, allowing controlled, repeatable low-frequency loading of the sample. The open-circuit voltage generated by the hybrid composite in response to the applied mechanical excitation was measured in real time using the oscilloscope, enabling characterization of the electromechanical conversion efficiency across different loading frequencies.

### 4.9 | Ultrasonic Powering

Ultrasonic powering of hybrid composites was carried out using a focused ultrasonic transducer (TXH- 0.67–75, Precision Acoustics; *f*_resonant_ = 670 kHz) as the transmitter. The ultrasonic transducer was driven via a function generator (33500b, Keysight) and a power amplifier (1040L, ENI Inc.). The generated ultrasonic waves traveled through the water medium and excited the hybrid composite disc, which was affixed to a shaft at the focal distance from the transmitter. The electrical output obtained from the hybrid composite discs was captured using an oscilloscope.

### 4.10 | Biocompatibility Assessment

The biocompatibility of BTO-YSZ composites was evaluated using extract-based cytotoxicity assays in accordance with ISO 10993–5:2009 guidelines. Human gingival keratinocytes (HGKs) and human gingival fibroblasts (HGFs) were used for the assay. HGKs were obtained from the laboratory of Dr. Dana T. Graves, and HGFs from the laboratory of Dr. Jonathan Korostoff, both at the School of Dental Medicine, University of Pennsylvania. Cells were seeded in 96-well plates at a density of 5000 cells/cm^2^ and cultured in their respective media. HGKs were cultured in Keratinocyte Basal Medium (KBM-Gold, Lonza, Basel, Switzerland) supplemented with the KGM-2 SingleQuots Kit (Lonza), while HGFs were maintained in Fibroblast Basal Medium (ATCC, Manassas, VA) supplemented with the Fibroblast Growth Kit (ATCC, PCS-201-041). Cells were incubated at 37° C in a humidified atmosphere with 5% CO_2_ until they reached monolayer confluence. BTO–YSZ discs at varying mixing ratios, as well as control samples, were incubated in culture media at 3 cm^2^/mL (surface area/volume ratio) for 24 h to generate extracts. After the cells reached confluency, the culture media in each well were replaced with the respective extracts and incubated for an additional 24 h. The next day, the extract media was removed and replaced with fresh culture media. To assess metabolic activity, 10 μL of MTT reagent (Sigma–Aldrich, St. Louis, MO) was added to 90 μL of fresh, serum-free media and incubated for 5 h. The supernatant was then carefully removed, and dimethyl sulfoxide (DMSO; Sigma–Aldrich) was added to dissolve the resulting formazan crystals. Absorbance was measured at 570 nm using a SpectraMax M2 microplate reader (Molecular Devices, Sunnyvale, CA). Cell viability was calculated based on absorbance values, normalized to the untreated control.

### 4.11 | Cell Adhesion Assay

To examine cell adhesion on BTO–YSZ surfaces, discs of varying compositions were first cleaned with Milli-Q water to remove surface debris and then air-dried. Samples were sterilized using UV light for 15 min on each side before being placed in a 96-well plate. HGKs and HGFs were seeded directly onto the sample surfaces at a density of 4 × 10^4^ cells per pellet and incubated 24 h under standard culture conditions. Following incubation, cells were fixed with 4% paraformaldehyde and subjected to staining protocols to visualize adhesion. Cell morphology and surface attachment were assessed using a Keyence fluorescence microscope at 4x and 10x magnification.

## Supplementary Material

Supplementary material

Additional supporting information can be found online in the Supporting Information section.

**Supporting File:** smll73130-sup-0001-SuppMat.docx.

## Data Availability

The data that support the findings of this study are available from the corresponding author upon reasonable request.
