# Supplementary material for: Hybrid Bioceramic: A Synergistic Platform for Structural Reinforcement and Bioelectric Stimulation Toward Smart Medical Devices
Source: Small. Author manuscript; Available in PMC 2026 Jun 5. (PMC13241089; doi:10.1002/smll.202513459)
Supplement: Supplementary material [file NIHMS2177035-supplement-Supplementary_material.docx]

**Supporting Information**

**Hybrid Bioceramic: A Synergistic Platform for Structural Reinforcement and Bioelectric Stimulation toward Smart Medical Devices**

*Sophia Selvarajan, Eunjung Byun, Md Naimur Rahman Niloy, Junjira Tanum, Heejin Hwang, Seung Hyun Song, Geelsu Hwang, and Albert Kim**

S. Selvarajan, M. Niloy, A Kim

Department of Medical Engineering

College of Engineering

University of South Florida

Tampa, FL, 33620, USA

E-mail: akim1@usf.edu

E. Byun, H. Hwang, S. Song

Department of Electrical Engineering

College of Engineering

Sookmyung Women’s University

Seoul, 04312, Republic of Korea

J. Tanum, G. Hwang

Department of Preventive and Restoration Sciences

School of Dental Medicine

University of Pennsylvania

Philadelphia, PA, 19104, USA

Funding:

US NSF: ECCS-2245090, ECCS-2300985/2225697, BMAT-2321385/2321384.

US NIH: R01DE32343, R21DE32162.

US FLDoH: MOADB.

S. Korea: MIST: RS-2024-00357197, RS-2024-00347107.

Keywords: barium titanate, zirconia, piezoelectric, implantable, structural integrity

**Fabrication process of BTO–YSZ hybrid material:**

**
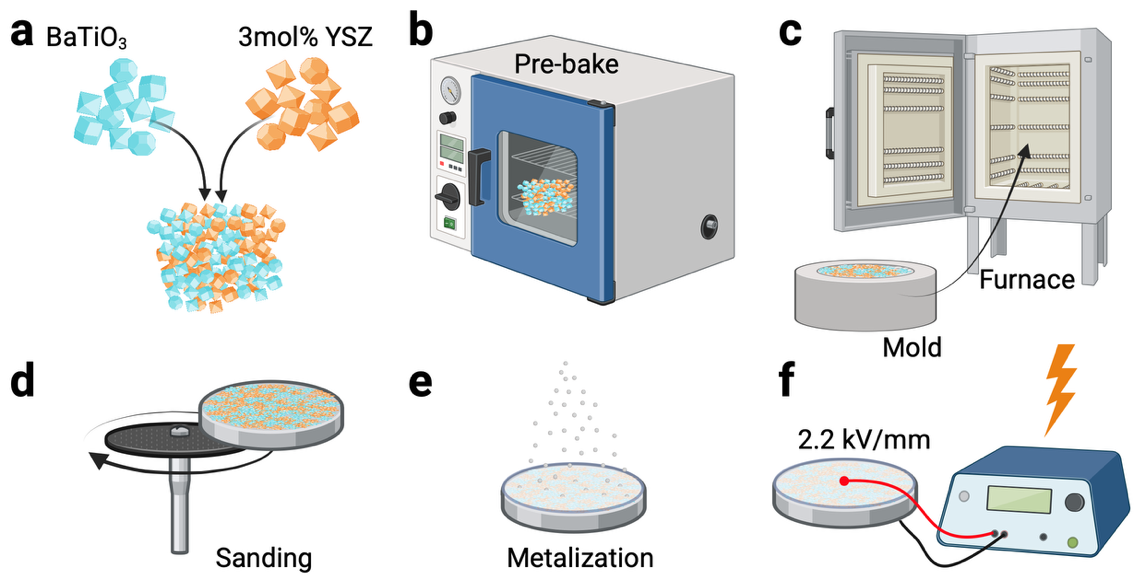
**

**Figure S1.** Fabrication process of BTO–YSZ composites: a) Mixing BTO and YSZ at preset ratios (i.e., 40:60, 50:50, 60:40), b) pre-bake at 100°C, c) the mixture of BTO–YSZ is packed in wax resin mold and sintered at desired temperature (i.e., 1,250 – 1,450 °C). d) The sintered samples are polished to reduce surface roughness, e) followed by silver metalization. f) The process is completed by the poling by ~2.2 kV/mm.

**SEM Microstructure of Pristine BTO and YSZ Ceramics:**


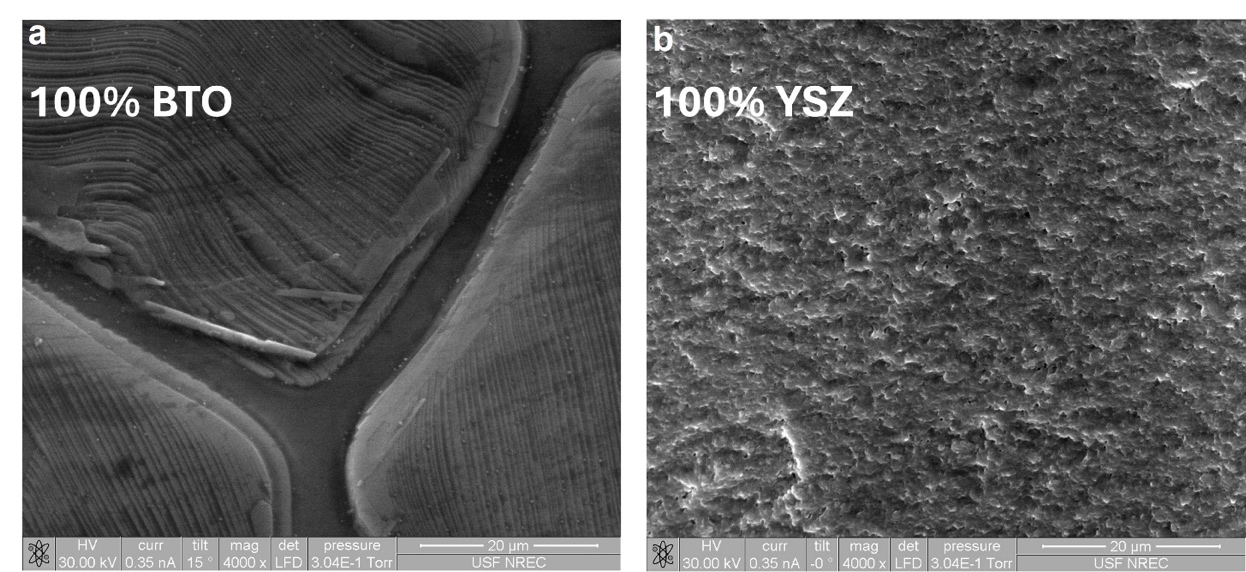


**Figure S2. Representative SEM micrographs** of (a) 100% BTO and (b) 100% YSZ ceramics sintered at 1,350 °C, imaged at 4000× magnification. The images illustrate distinct microstructural characteristics of each phase prior to composite formation.

The SEM image of pure BTO (Figure S1a) shows large, plate-like grains with terraced surfaces and clear grain boundaries—features typical of ferroelectric perovskite ceramics. These structures support domain wall motion, which is essential for strong piezoelectric performance. In contrast, the 100% YSZ sample (Figure S1b) displays a uniform, fine-grained microstructure with no visible faceting, as expected for fully stabilized zirconia. This morphology suggests better mechanical toughness but limited piezoelectric activity. Together, these baseline images support the strategy of combining BTO and YSZ to achieve a balance between electrical functionality and mechanical durability in composite systems.

**Densification Behavior of BTO–YSZ Composites**

The density of the composite samples increased with sintering temperature for all compositions, indicating progressive densification. The 50:50 composition achieved the highest density (~5.2 g/cm³) at 1450 °C, suggesting optimal packing and grain boundary consolidation. The 60:40 and 40:60 compositions followed a similar trend but plateaued at lower density values. The enhanced densification in 50:50 can be attributed to balanced phase distribution and minimal porosity, while the 40:60 samples may exhibit restricted grain growth due to higher YSZ content. These observations are consistent with the observed mechanical integrity in later tests.


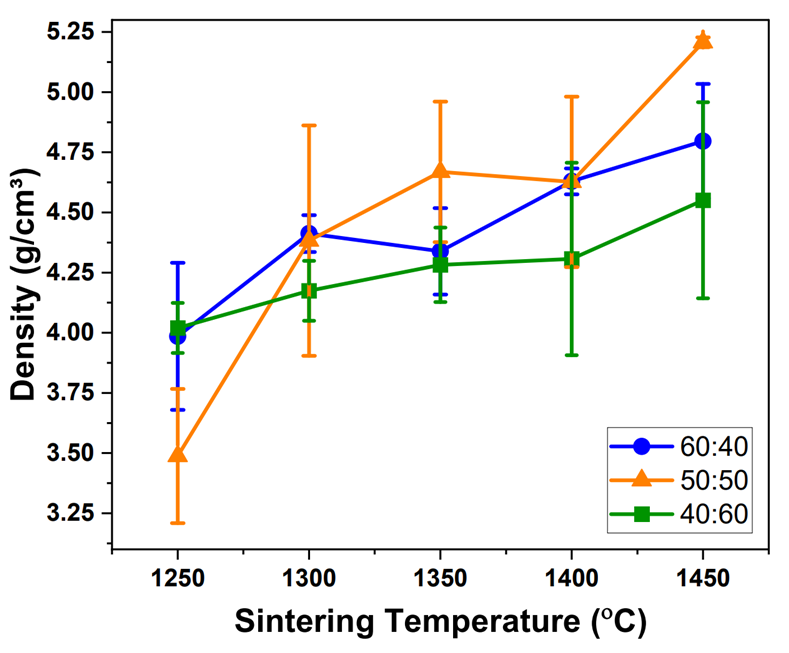


**Figure S3.** **Sintering temperature-dependent variation in the density of BTO–YSZ composites** for three different compositions: 60:40, 50:50, and 40:60 (BTO:YSZ). Error bars represent the standard deviation from multiple measurements.

**Electromechanical Response under Vibration Excitation**

Electromechanical output improved with increasing excitation frequency for all compositions. The 50:50 composite sintered at 1,350 °C displayed the highest output of ~1.7 V at 10 Hz, due to optimal domain alignment and material densification. The 60:40 composition also exhibited a high voltage output (~1.2 V) at similar sintering temperatures. However, 40:60 composites consistently produced lower voltages (~500–750 mV), which may be attributed to the suppression of ferroelectric domain formation due to excess YSZ. The peak performance at 1,350 °C aligns with optimal grain growth before the onset of coarsening or secondary phase formation at higher temperatures.


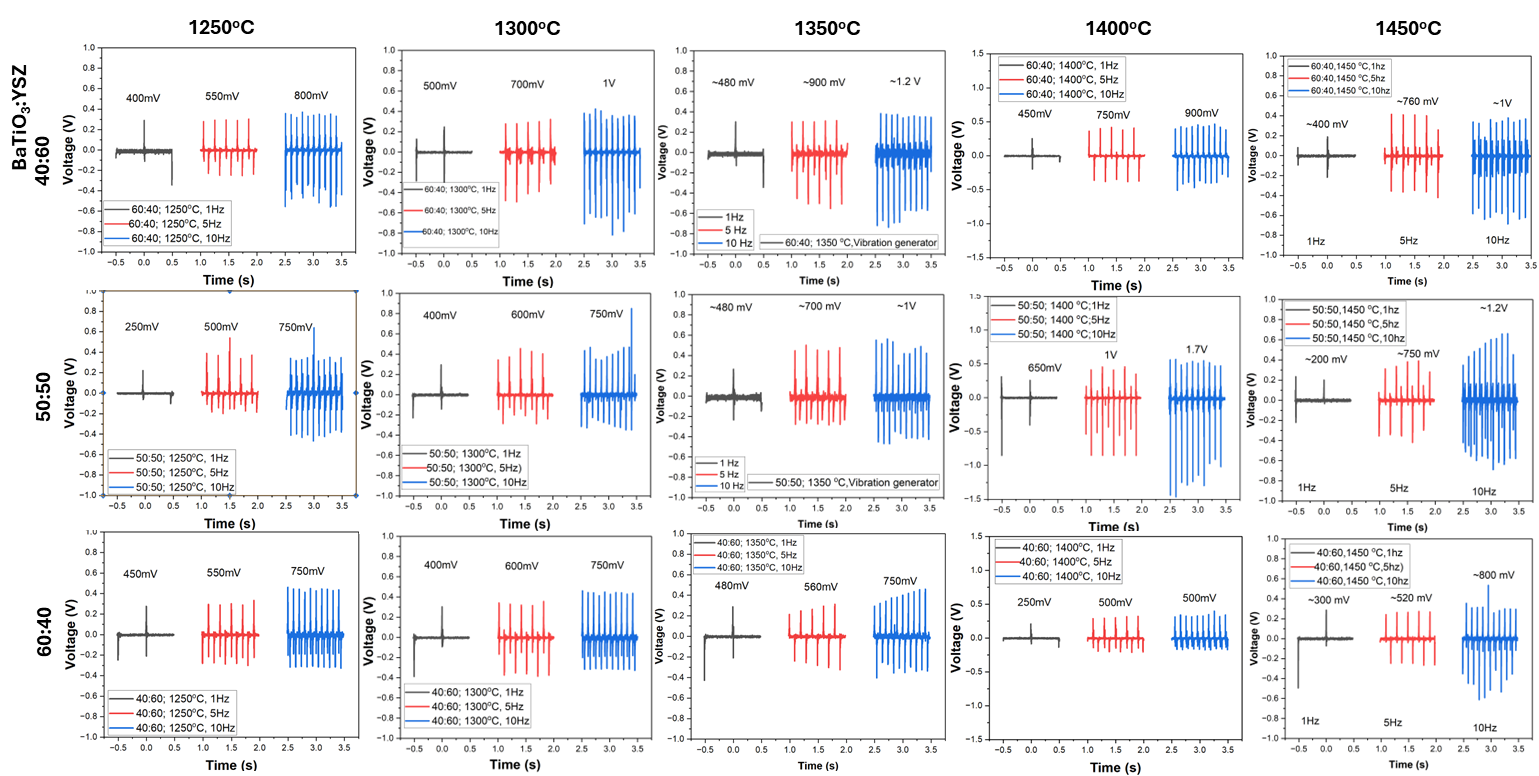


**Figure S4. Voltage output response of BTO–YSZ** **composites** sintered at various temperatures under mechanical excitation at 1 Hz, 5 Hz, and 10 Hz using a vibration generator. Each row corresponds to a different composition: 60:40 (top), 50:50 (middle), and 40:60 (bottom). Voltage output increases with excitation frequency and sintering temperature.

**Voltage Output under Thumb Press Stimulation**

Under thumb press conditions, the voltage output ranged from ~160 mV to ~460 mV depending on composition and sintering temperature. The highest output (~460 mV) was observed for the 60:40 sample, which was sintered at 1400 °C. These results confirm that the composites are sensitive to low-frequency, low-force mechanical stimuli—relevant for applications such as smart dental implants and wearable sensors. Compared to dynamic excitation (Fig. S2), thumb press responses are lower in magnitude but consistent across replicates, reflecting material robustness and surface response uniformity. The gradual increase in output with sintering temperature is likely due to improved grain connectivity and poling efficiency.


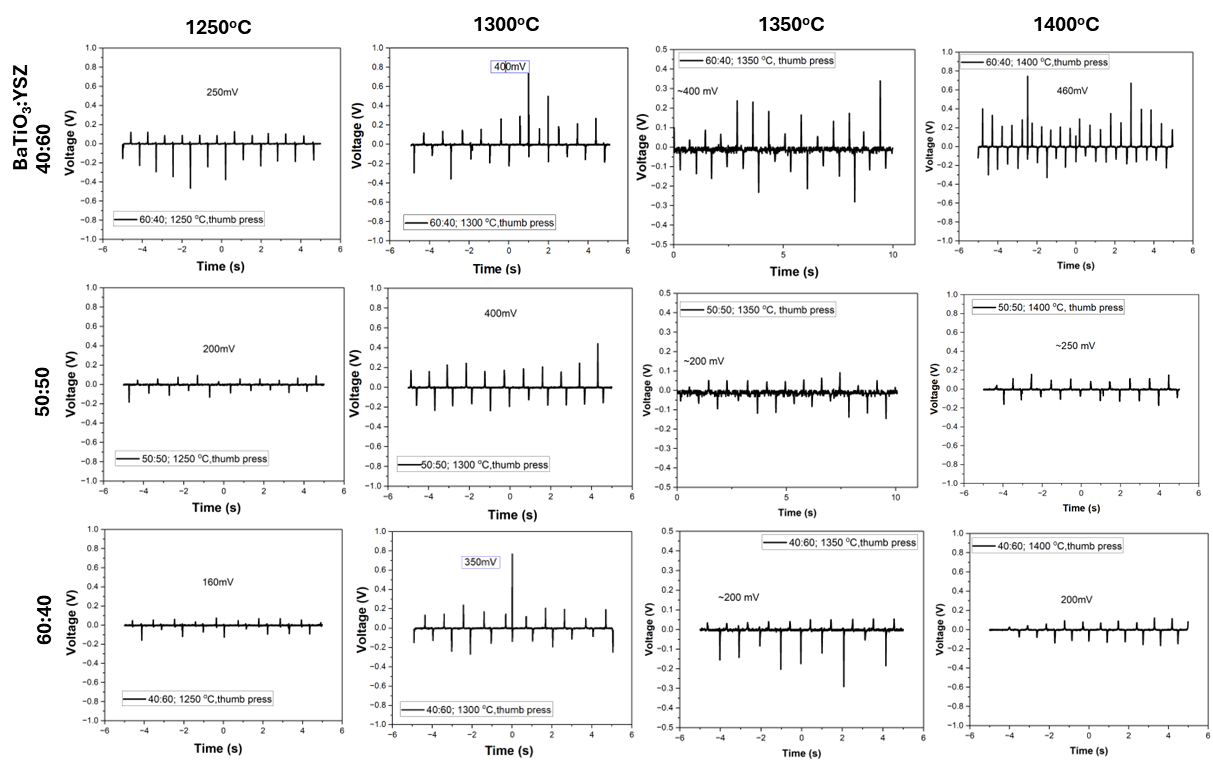


**Figure S5. Open-circuit voltage output of BTO–YSZ composites in response to manually applied thumb pressure.** Rows correspond to different compositions: 60:40 (top), 50:50 (middle), and 40:60 (bottom), across various sintering temperatures.

**Polarization Condition**


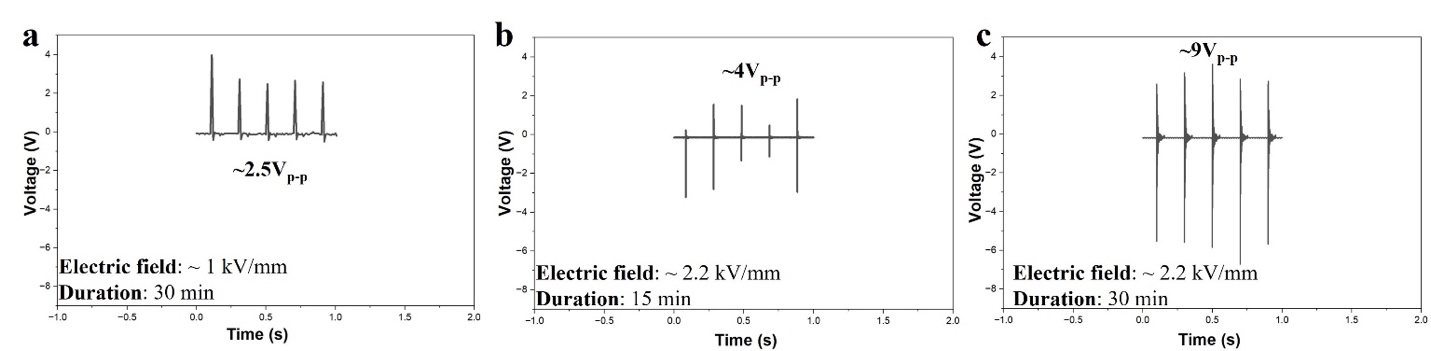


**Figure S6.** **Optimization of poling conditions using 100% BaTiO₃.** Representative voltage outputs obtained after poling under different electric field strengths and durations: (a) ~1 kV mm⁻¹ for 30 min, (b) ~2.2 kV mm⁻¹ for 15 min, and (c) ~2.2 kV mm⁻¹ for 30 min. Increasing electric field strength and poling duration leads to progressively enhanced piezoelectric output.


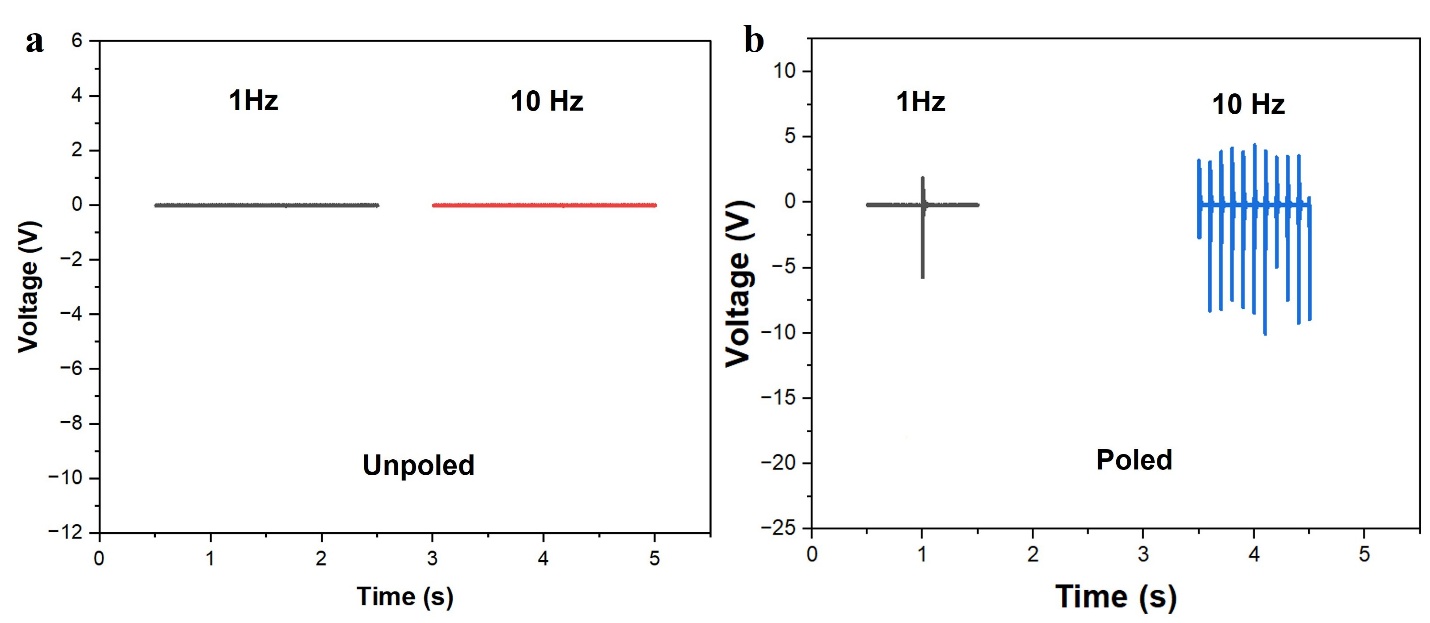


**Figure S7.** Comparison of electromechanical response of 100% BaTiO₃ before and after poling under low-frequency mechanical excitation. (a) Unpoled sample showing negligible voltage output at 1 Hz and 10 Hz, and (b) poled sample exhibiting a pronounced and stable voltage response under identical excitation conditions.
